# Statistical Behaviors of Semiflexible Polymer Chains Stretched in Rectangular Tubes

**DOI:** 10.3390/polym11020260

**Published:** 2019-02-04

**Authors:** Jizeng Wang, Kai Li

**Affiliations:** Key Laboratory of Mechanics on Disaster and Environment in Western China, Ministry of Education, College of Civil Engineering and Mechanics, Lanzhou University, Lanzhou 730000, Gansu, China; kli2015@lzu.edu.cn

**Keywords:** wormlike chain model, rectangular tube confinement, slit confinement, Odijk length, stretch, GBR model, Brownian dynamics simulation

## Abstract

We investigated the statistical behaviors of semiflexible polymer chains, which were simultaneously subjected to force stretching and rectangular tube confinement. Based on the wormlike chain model and Odijk deflection theory, we derived a new deflection length, by using which new compact formulas were obtained for the confinement free energy and force–confinement–extension relations. These newly derived formulas were justified by numerical solutions of the eigenvalue problem associated with the Fokker–Planck governing equation and extensive Brownian dynamics simulations based on the so-called generalized bead-rod (GBR) model. We found that, compared to classical deflection theory, these new formulas were valid for a much more extended range of the confinement size/persistence length ratio and had no adjustable fitting parameters for sufficiently long semiflexible chains in the whole deflection regime.

## 1. Introduction

Statistical physics properties of single polymer chains can be significantly influenced or even determined by geometrical confinements and applied external forces [1,2,3,4]. A detailed understanding of the behaviors of polymers under such circumstances is still considered to be an unsolved problem in polymer physics after more than half a century. However, even so, advances in the study of geometrically and potentially constrained polymers are playing a role in the development of many existing nanotechnologies of genomics and materials science, etc. [5,6,7].

For polymers under confinement, the effects of constraints have usually been classified into three regimes (weak, moderate, and strong confinements), which are distinguished in terms of the comparison between the polymer’s unconfined radius of gyration and the Kuhn length of the typical confinement length scale. In the regime of weak confinement, Casassa [8] has discussed the free energy of ideal chains trapped in pores with different shapes based on the theory of diffusion. Then, de Gennes and his coworkers [9,10] developed the so-called blob model to describe the confinement of a long, flexible polymer. According to this model, the free energy of confinement is given by

(1) $$\frac{F}{k_{B}T}\propto {\left(\frac{R_{g}}{H}\right)}^{1/v}.$$

Here, *H* is the length scale of confinement, $k_{B}$ is the Boltzmann constant, and *T* is the absolute temperature. $R_{g}\approx L_{a}N^{v}$ is the radius of gyration of the unconfined polymer, *N* is the number of monomers, $L_{a}$ is of the order of the monomer-monomer separation, and *v* equals 1/2 for ideal polymers and approximately 3/5 for polymers with excluded volume. A widely used model of a polymer with bending energy is the wormlike chain (WLC) model, characterized by the contour length *L* and persistence length *L*_p_, which was first proposed by Kratky and Porod in 1949 [11]. In the strong confinement regime, Odijk [12,13] has argued that polymer behavior can be interpreted in terms of L/λ statistically independent segments with deflection length λ. Based on this picture, he [12,13,14] obtained expressions of the confinement free energy, *F*, and the average extension of the chain, $R_{\left|\right|}$, in terms of λ as

(2) $$F\approx k_{B}T\frac{L}{\lambda_{\mathrm{fe}}},$$

(3) $$1-\frac{R_{∥}}{L}\approx \frac{\lambda_{\mathrm{ext}}}{2L_{p}},$$

where $\lambda_{\mathrm{fe}},\;\lambda_{\mathrm{ext}}$ represent the free energy and extension-associated deflection lengths, respectively: $\lambda_{\mathrm{fe}},\lambda_{\mathrm{ext}}\propto L_{p}^{1/3}D^{2/3}$ [12] has been suggested for the confinement of a cylindrical tube with diameter *D*. For the confinement of a rectangular tube with height and width *H*_h_ and *H*_w_, the deflection length associated with the free energy calculation has been suggested as [15,16]

(4) $$\lambda_{\mathrm{fe}}=\frac{1}{A_{□}}L_{p}^{1/3}{\left(H_{h}^{-2/3}+H_{w}^{-2/3}\right)}^{-1}.$$

In contrast, this deflection length associated with the average extension is given as

(5) $$\lambda_{\mathrm{ext}}=2\alpha_{□}(L_{p}^{1/3}H_{h}^{2/3}+L_{p}^{1/3}H_{w}^{2/3}).$$

We note that Equations (4) and (5) have different expressions and prefactors. The prefactors in Equations (4) and (5) were determined by using various numerical techniques and theoretical derivations, such as the Monte Carlo simulations [17,18] and eigenvalue technique associated with the Fokker–Planck equations [15,19]. Examples of the determined prefactors are illustrated in Table 1. We can see that the prefactors $1/A_{□}$ and $\alpha_{□}$, respectively determined from free energy and extension, have an almost 10 times difference in quantity.

In addition, a slit of separation *H* can be regarded as a rectangular tube with height *H*_h_ = *H* and infinite width $H_{w}^{}\to \infty$. Statistical properties of polymer chains confined in the slit have been extensively studied [20,21,22,23,24,25] based on Monte Carlo simulations and eigenvalue analysis. The deflection length in a strong confinement regime has been confirmed to follow the Odijk scaling law,

(6) $$\lambda \propto L_{p}^{1/3}H^{2/3}.$$

Although the deflection length in Equation (5) can be viewed as the combination of that for two slits with heights *H*_h_ and *H*_w_, respectively [15], it can be observed from Equations (4)–(6) that the deflection length in Equation (5) is not consistent with Equation (6) as $H_{w}^{}\to \infty$.

Beyond the Odijk regime, Chen [24] numerically calculated the confinement free energy by treating the problem of a confined polymer as an eigenvalue problem. He also suggested an interpolating formula that can have very good agreement with that of the numerical calculations for polymers under both strong and weak confinements. In addition, an extended de Gennes regime [20,26,27] has also been identified based on the Monte Carlo simulations.

Interestingly, external forces can pose similar effects to the statistical behaviors of single polymer chains as geometrical confinements. For a polymer chain to be stretched by a sufficiently large force, $f_{s}$, a deflection length also exists and can be expressed as [3,28] $\lambda_{f}=L_{p}/\sqrt{\hat{f}}$, where $\hat{f}=f_{s}L_{p}/k_{B}T$, so that the force-extension relation can be expressed as

(7) $$1-\frac{R_{∥}}{L}\approx \frac{1}{2\sqrt{\hat{f}}}.$$

Polymers in real microenvironments are usually subjected to both geometrical constraints and external forces. Wang and Gao [29] have revealed that the average extension of a strongly tube-confined and force-stretched polymer chain can be equivalent to that of an unconfined chain subjected to an effective stretching force. Li and Wang [30] later confirmed that this equivalence property is still valid for the tube-confined polymers in a much more extended Odijk regime. Therefore, for a semiflexible polymer chain in the deflection confinement regime, one can generally have

(8) $$1-\frac{R_{∥}}{L}\approx \frac{1}{2\sqrt{\hat{f}+{\hat{f}}_{c}}},$$

where one can set ${\hat{f}}_{c}=L_{p}^{2}/\lambda_{\mathrm{fe}}^{2}$ or ${\hat{f}}_{c}=L_{p}^{2}/\lambda_{\mathrm{ext}}^{2}$ as the normalized effective force due to the existence of strong confinement: For the confinements of rectangular tubes, $\lambda_{\mathrm{fe}}^{}$, $\lambda_{\mathrm{ext}}^{}$ are given by Equations (4) and (5).

However, as shown above, Odijk deflection lengths based on free energy and extension are different. Then a critical question arises. Which deflection length should be used if the polymer chain is under both geometrical confinement and force stretching? Therefore, there are still open questions on how the Odijk length can be uniquely and precisely defined for polymer chains confined in rectangular tubes.

In this study, for semiflexible polymer chains confined in rectangular tubes and slits, we derived a modified deflection length, which was expected to be valid for a more extended range than the classical Odijk deflection length. This extended deflection length was directly used to quantitatively formulate both energy and extension. We will present numerical calculations based on the eigenvalue technique developed by Chen and coworkers [19,31,32] and Brownian dynamics simulations in terms of the generalized bead-rod (GBR) model [30,33,34] to justify our theoretical predictions.

## 2. Materials and Methods

### 2.1. Model

**Figure 1:**
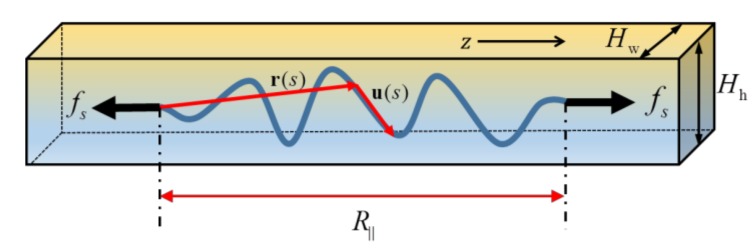
Schematic of a wormlike chain (WLC) confined in a rectangular tube and stretched by a force.

We first considered a WLC confined in a rectangular tube with width *H*_w_ and height *H*_h_, as shown in Figure 1. We assumed that the tube was small so that the chain’s configurations with the so-called “hairpin” structures [14,35] rarely existed. This means statistical behaviors of the chain fell into the deflection regime. In order to obtain a universal deflection length scale, *λ*, to simultaneously characterize both the free energy and extension of the chain in terms of the ideas of de Gennes [9,10] and Odijk [12], the chain was assumed to behave like *L*/*λ* independent free segments, aligning one by one along the tube axis, so that the conformational free energy could still be scaled as Equation (1) and the average extension could be simply the sum of that for each free segment. On the other hand, when considering the average extension of the chain, the quantitative behavior of each segment should be in analogy with a free chain of effective contour length $c_{1}\lambda_{m}$, in which the parameter *c*_1_ actually reflects the influence of two artificial ends of each such segment. For a free WLC segment of contour length $c_{1}\lambda_{m}$, projection of the position vector of one end, **r**(*s*_2_), to the tangential vector of the other end, **u**(*s*_1_), can be given by [36]

(9) $$\langle r(s_{1})\cdot u(s_{2})\rangle =L_{p}(1-e^{-c_{1}\lambda_{m}/L_{p}}).$$

Then the average extension of the whole chain can be estimated as

(10) $$R_{\left|\right|}=c_{2}\frac{L}{\lambda_{m}^{}}\langle r(s_{1})\cdot u(s_{2})\rangle =c_{2}\frac{L_{p}L}{\lambda_{m}}(1-e^{-c_{1}\lambda_{m}/L_{p}}),$$

in which $c_{2}$ is introduced as an unknown dimensionless factor. Equation (10) should reproduce Equation (3) in the deflection regime, which can determine $c_{1}=\vartheta =8A_{□}\alpha_{□}$ and $c_{2}=1/\vartheta$, so that eventually we have

(11) $$R_{\left|\right|}=\frac{LL_{p}}{\vartheta \lambda_{m}}\left(1-e^{-\vartheta \lambda_{m}/L_{p}}\right).$$

For a tightly confined polymer in a channel with a rectangular cross-section, Burkhardt and Yang et al. [16,19] have derived that the confinement free energy of the polymer chain can be scaled by the average length of the tube occupied by the polymer, which is the average extension of the polymer chain, as follows:

(12) $$\frac{F}{R_{\left|\right|}}=A_{□}\frac{k_{B}T}{L_{p}^{1/3}}(H_{h}^{-2/3}+H_{w}^{-2/3}).$$

Assuming that $\lambda_{m}$ should satisfy Equations (2), (11), and (12), one has

(13) $$\frac{L}{\lambda_{m}}k_{B}T=A_{□}\frac{k_{B}T}{L_{p}^{1/3}}(H_{h}^{-2/3}+H_{w}^{-2/3})\frac{LL_{p}}{\vartheta \lambda_{m}}\left(1-e^{-\vartheta \lambda_{m}/L_{p}}\right),$$

or

(14) $$\frac{\lambda_{m}}{L_{p}}=-\frac{1}{\vartheta }\ln[1-\vartheta A_{□}^{-1}{\left({\hat{H}}_{h}^{-2/3}+{\hat{H}}_{w}^{-2/3}\right)}^{-1}],$$

where ${\hat{H}}_{h}^{}≜H_{h}^{}/L_{p}$ and ${\hat{H}}_{w}^{}≜H_{w}^{}/L_{p}$. Equation (14) can be regarded as a new deflection length that fulfills both requirements for the free energy and statistics of geometrical quantities. We can see from Equation (14) that as long as $\mathrm{Min}\left(H_{w},\;\;H_{h}\right)/L_{p}\ll 1$, Taylor expansion of this equation yields the result in Equation (4). Inserting Equation (14) into Equation (2), and replacing $\lambda_{\mathrm{fe}}$ by $\lambda_{m}$, we can obtain the confinement free energy as follows:

(15) $$\frac{F}{k_{B}T}=-\frac{\vartheta L}{L_{p}\ln[1-\vartheta A_{□}^{-1}{\left({\hat{H}}_{h}^{-2/3}+{\hat{H}}_{w}^{-2/3}\right)}^{-1}]}.$$

For the extension of the chain under both confinement and stretching force, as shown in Figure 1, Wang and Li [37] have suggested the force-extension relation shown in Equation (8), which now can be rewritten as

(16) $$1-\frac{R_{∥}}{L}\approx \frac{1}{2}\frac{1}{\sqrt{\hat{f}+{\left\{-\ln[1-\vartheta A_{□}^{-1}{\left({\hat{H}}_{h}^{-2/3}+{\hat{H}}_{w}^{-2/3}\right)}^{-1}]\right\}}^{-2}}}.$$

On the other hand, substituting Equation (5) into Equation (3), we can obtain the classical extension relation without stretching in the tight-confinement regime, $H_{h}/L_{p}\ll 1$, $H_{w}/L_{p}\ll 1$, as follows:

(17) $$1-\frac{R_{∥}}{L}\approx \alpha_{□}({\hat{H}}_{h}^{2/3}+{\hat{H}}_{w}^{2/3}).$$

In the case of $\hat{f}=0$, Equation (16) in this regime can be reduced to

(18) $$1-\frac{R_{∥}}{L}\approx 4\alpha_{□}{\left({\hat{H}}_{h}^{-2/3}+{\hat{H}}_{w}^{-2/3}\right)}^{-1}.$$

Equations (17) and (18) clearly agree in the special case ($H_{h}=H_{w}$) of a tube with a square cross-section. In terms of the aspect ratio $\beta =H_{h}^{}/H_{w}^{}$, the right-hand side of Equation (17) is larger than the right-hand side of Equation (18) by the factor

(19) $$\phi \left(\beta \right)=\frac{1}{4}{\left(\beta^{1/3}+\beta^{-1/3}\right)}^{2}.$$

The function ϕ(β) has a single minimum at β=1, corresponding to ϕ(1)=1, and approaches ∞ in the limits β→0 and β→∞. Thus, Equation (18) only agrees with the classical strong confinement limit (17) if the cross-section of the tube is square or close to it.

### 2.2. Numerical Verifications

#### 2.2.1. Solutions to the Fokker–Planck Equation

In order to verify the derived free energy expression, we considered the solutions to the Fokker–Planck equation, which can be used to describe the statistical behaviors of confined polymer chains. We first introduce q(r,u,s) to represent the probability that a polymer chain at arc length *s* has an end position vector **r** and an end unit tangential vector **u**. Then, we can have the partition function of the chain with contour length *L* as $Z=\int^{\text{​}}drduq\left(r,u,L\right)$ and the Fokker–Planck equation [31,32]

(20) $$\frac{\partial }{\partial s}q(r,u,s)=\left\{-u\cdot \nabla_{r}+\frac{1}{2L_{p}}\nabla_{u}{}^{2}+\left[\left(u\cdot \nabla_{r}\right)u\right]\cdot \nabla_{u}-\frac{1}{k_{B}T}V(r)\right\}q(r,u,s),$$

where

(21) $$V(r)=\{\begin{array}{cc} 0, & |x|<H_{w}/2\;\mathrm{and}\;\;|y|<H_{h}/2\\ \infty , & \mathrm{Otherwise} \end{array}$$

is the potential energy per unit length due to the confinement of a rectangular tube. As suggested by Chen [19,32], the solution of Equation (20) can be expanded into a series of eigenfunctions associated with negative exponential terms of eigenvalues. By noting that the chain is sufficiently long and that the high-order eigenvalues are large enough, then the solution can be approximated by the ground state eigenfunction $\Psi_{0}(r,u)$ and eigenvalue $\mu_{0}$ as follows:

(22) $$q(r,u,L)\approx \exp(-\frac{\mu_{0}L}{2L_{p}})\Psi_{0}(r,u).$$

Then the free energy can be written as

(23) $$F=-k_{B}T\ln Z\approx k_{B}TL\frac{\mu_{0}}{2L_{p}}.$$

Comparing Equation (2) to Equation (23) gives

(24) $$\lambda \;=\frac{2L_{p}}{\mu_{0}}.$$

**Figure 2:**
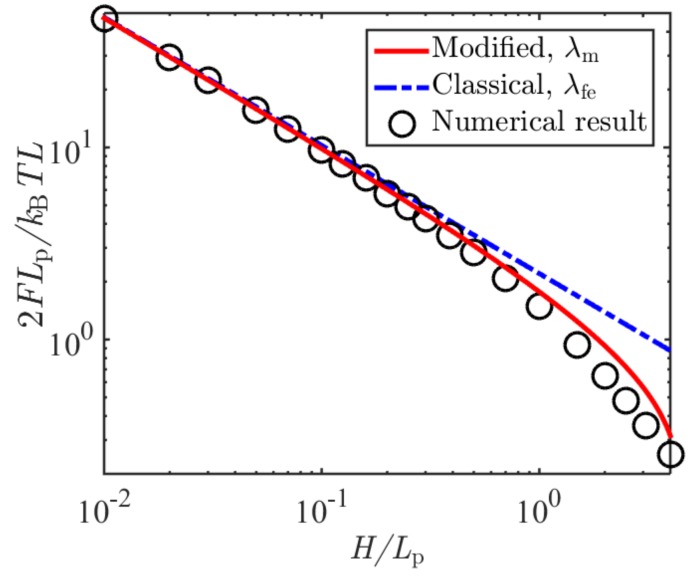
Normalized confinement free energy as a function of the normalized slit height, predicted based on the classical and modified deflection lengths, and the solutions of the eigenvalue problem.

Chen and his coworkers [19,31,32] have proposed an iteration method to numerically determine the ground state eigenvalue and eigenfunction. In this study, we adopted this method to calculate the confinement free energy. As examples, we considered polymer chains confined in slits with different heights *H*. When using Chen’s method to calculate $\mu_{0},$ we set the tolerance error as $10^{-4}$. Figure 2 shows the comparison of the confinement free energy as a function of *H/L*_p_ obtained by numerical solutions of the ground state eigenvalue, Equation (15), and Equation (2) in terms of the classical Odijk length. It can be seen from Figure 2 that free energy based on the modified deflection length had a better agreement with the numerical results than that based on the classical one.

#### 2.2.2. Brownian Dynamics Simulations (Appendix A)

We used the technique of statistical dynamics simulations to verify the derived force-extension relation on polymer chains subjected to both confinement of rectangular tubes and stretching of external forces. We performed the simulations by using our GBR model for Brownian dynamics of semiflexible polymer chains in confinements [33]. This model has been successfully applied to the quantitative analysis of statistical behaviors of polymers confined on spherical surfaces [34] and in cylindrical tubes [29,30] and subjected to stretching forces [29,30]. In this GBR model, we considered the polymer chain as a discrete WLC with *N* identical virtual beads of radius *a* at different positions $r_{k}(t)=\{x_{k}(t),y_{k}(t),z_{k}(t)\}\prime$}, where *k* = 1, 2, …, *N*, linked by *N*-1 rods with inextensible length *b*. The virtual beads are used to feel the hydrodynamic interactions, and angle changes of the adjacent rods are used to account for the bending deformation. As long as the position vectors of all *N* beads at the *n*th time step, denoted as $r_{\left(n\right)}$ = {$r_{1,(n)}$, $r_{2,(n)}$, …, $r_{N,(n)}$}’, are obtained, the new position vector at the (*n* + 1)th time step, $r_{\left(n+1\right)}$, can be calculated from [33,34]

(25) $$r_{\left(n+1\right)}=(I-T_{\left(n\right)}B_{\left(n\right)})(r_{\left(n\right)}+\chi_{\left(n\right)}^{\mathrm{wall}}+\frac{\Delta t}{k_{B}T}D_{\left(n\right)}F_{\left(n\right)}+\xi_{\left(n\right)})+T_{\left(n\right)}d,$$

where Δt is the time step, $\delta_{nn\prime }$ is the Kronecker delta symbol, $F_{\left(n\right)}$ is the collective vector of internal and external forces, $I-T_{\left(n\right)}B_{\left(n\right)}$ is a projection matrix (which together with $T_{\left(n\right)}d$ sets the inextensible constraints), $\chi_{\left(n\right)}^{\mathrm{wall}}$ is the penalty displacement vector for the tube/slit walls, $D_{\left(n\right)}$ is the translational diffusion matrix determined through hydrodynamic interactions between beads, and $\xi_{\left(n\right)}$ is the vector of random force generated at each time step from a Gaussian distribution with zero mean and variance equal to

(26) $$\langle \xi_{\left(n\right)}\xi_{\left(n\prime \right)}\rangle =2D_{\left(n\right)}\Delta t\delta_{nn\prime }.$$

We performed Brownian dynamics simulations for WLCs confined in square tubes, rectangular tubes, and narrow slits of different sizes and subjected to various stretching forces. In all simulations, the chains were initially set in a straight configuration. Confinements and constant tensile forces were then applied during chain relaxation. At the *n*th time step, we recorded the end-to-end distance along the *z* axis, $z_{N,\left(n\right)}-z_{0,\left(n\right)}$. For each simulation, we needed to keep the steady extension states lasting a sufficiently long enough time to generate enough numbers of different equilibrium configurations. For each case, average extension of the WLC was obtained by first averaging over time and then averaging over a large number of independent trajectories with different random seeds, which was then denoted as $R_{\left|\right|}$. For the simulation parameters, we should note that the contour length should be larger than (at least) two times the persistence length and much larger than the deflection length scale $\lambda_{m}$. As we were only interested in the equilibrium properties of the polymer chains, therefore specific values of the bead radius, the hydrodynamic interaction between beads, and time steps were not the key factors as long as sufficiently large numbers of different configurations of the polymer chain could be generated for averaging. In addition, the bond length should be selected to be much smaller than the deflection length scale $\lambda_{m}$ and the persistence length. For all these Brownian dynamics simulations, we chose persistence length of the chain as *L*_p_ = 50 nm, the viscosity of water as $\eta_{0}=1.005725\times 10^{-4}\;\mathrm{Pa}\cdot s$, and the absolute temperature as T=293 K. Simulation parameters on bead radius *a*, bond length *b*, time step Δt, contour length *L*, total simulation time, and total number of different trajectories for the ensemble average are listed in Table 2, Table 3, Table 4, Table 5 and Table 6 for different chains in different confinements.

**Figure 3:**
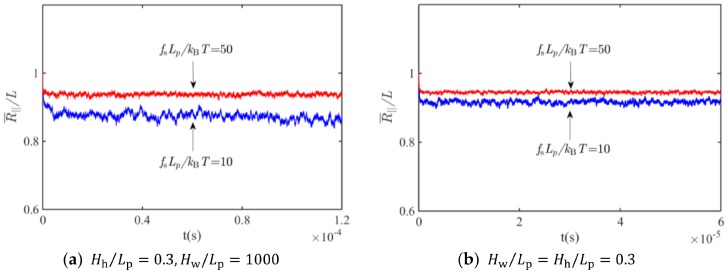
Evolution of the extension for tube-confined WLCs under stretching: (**a**) Slit, $H_{h}/L_{p}=0.3,\;\;H_{w}/L_{p}=1000$; (**b**) Square tube,$\;H_{w}/L_{p}=H_{h}/L_{p}=0.3$.

Figure 3 shows convergence of the simulations for the evolution of the ensemble average of the extension, ${\bar{R}}_{\left|\right|}$, for slit- and square tube-confined WLCs under stretching. It can be seen from Figure 3 that the equilibrium state could last a sufficiently long enough time to guarantee the effectiveness of time averaging.

**Figure 4:**
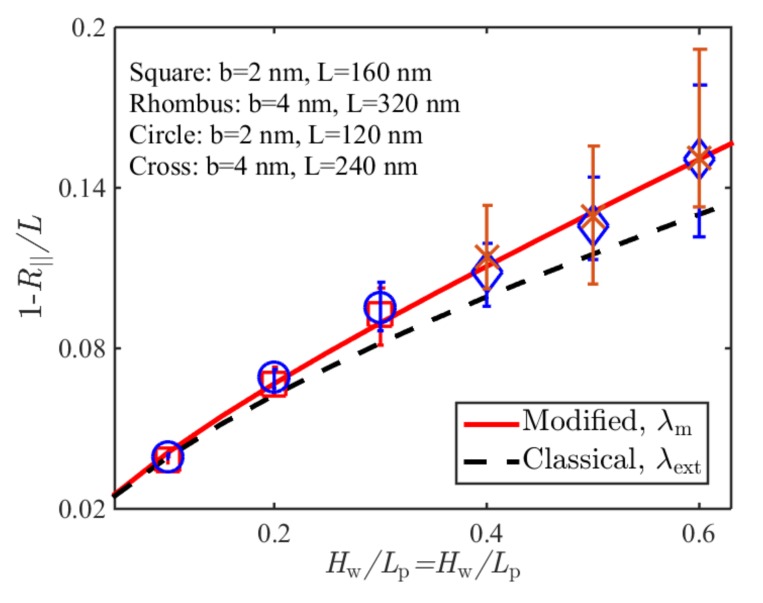
Comparison of Brownian dynamics simulations and theoretical predictions on the relative average extension of a WLC under confinements of square tubes.

Figure 4 shows the comparison of Brownian dynamics simulation results and corresponding theoretical predictions based on the classical deflection length as shown in Equation (17) and based on the modified deflection length in Equation (16) for the normalized average extension of the WLCs confined in square tubes of different sizes without stretching. Simulation parameters are listed in Table 2. It can be seen from Figure 4 that the prediction in terms of the modified deflection length agreed well with the simulation results, and could be reduced to that of the classical one at a tight confinement limit. For the case of a large *H*_w_/*L*_p_, the prediction in terms of the classical deflection length exhibited a large discrepancy with the simulation results.

**Figure 5:**
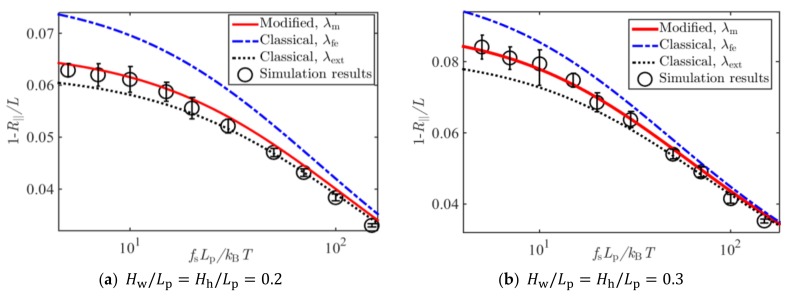

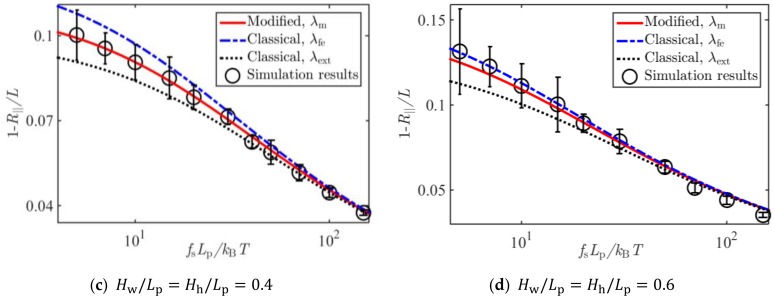
Comparison of Brownian dynamics simulations and theoretical predictions on the relative average extension of a WLC under stretching and confinements of square tubes of sizes (**a**) $H_{w}/L_{p}=H_{h}/L_{p}=0.2$, (**b**) $H_{w}/L_{p}=H_{h}/L_{p}=0.3$, (**c**) $H_{w}/L_{p}=H_{h}/L_{p}=0.4$, and (**d**) $H_{w}/L_{p}=H_{h}/L_{p}=0.6$.

**Figure 6:**
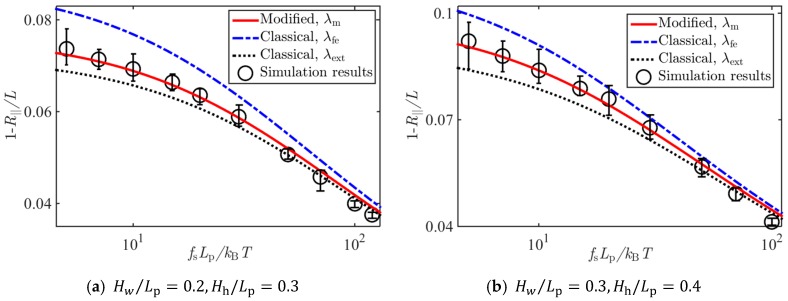

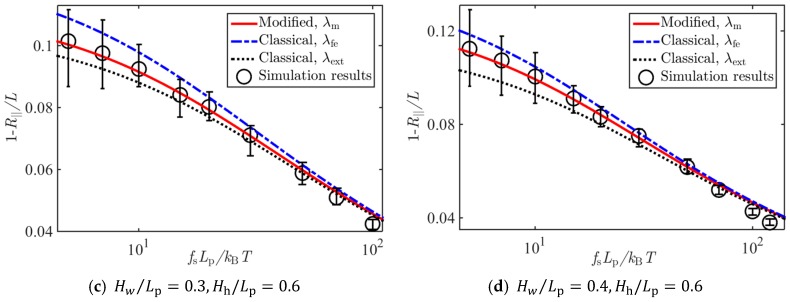
Comparison of Brownian dynamics simulations and theoretical predictions on the relative average extension of a WLC under stretching forces and confinements of rectangular tubes with sizes (**a**) $H_{w}/L_{p}=0.2,H_{h}/L_{p}=0.3$, (**b**) $H_{w}/L_{p}=0.3,H_{h}/L_{p}=0.4$, (**c**) $H_{w}/L_{p}=0.3,H_{h}/L_{p}=0.6$, and (**d**) $H_{w}/L_{p}=0.4,H_{h}/L_{p}=0.6$.

**Figure 7:**
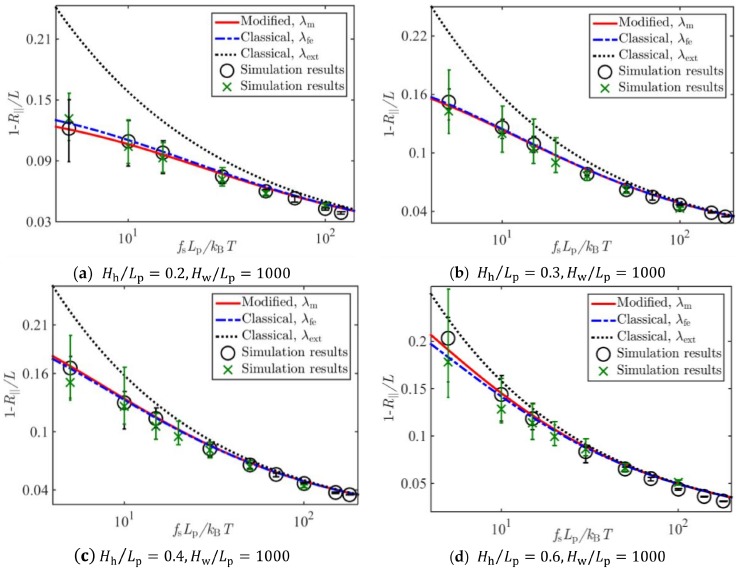
Comparison of Brownian dynamics simulations and theoretical predictions on the relative average extension of a WLC under stretching forces and confinements of slits with sizes (**a**) $H_{h}/L_{p}=0.2,H_{w}/L_{p}=1000$, (**b**) $H_{h}/L_{p}=0.3,H_{w}/L_{p}=1000$, (**c**) $H_{h}/L_{p}=0.4,H_{w}/L_{p}=1000$, and (**d**) $H_{h}/L_{p}=0.6,H_{w}/L_{p}=1000$.

Figure 5, Figure 6 and Figure 7 show the comparison of Brownian dynamics simulation results and corresponding theoretical predictions, based on Equation (8), associated with the classical deflection lengths $\lambda_{\mathrm{fe}}$ in Equation (4), $\lambda_{\mathrm{ext}}$ in Equation (5), and that based on Equation (17) associated with the present new deflection length $\lambda_{m}$ in Equation (16), for the normalized average extension of the WLCs stretched by different forces and confined in square tubes, rectangular tubes, and slits of different sizes, respectively.

It can be seen from Figure 5, Figure 6 and Figure 7 that results based on the newly derived formula on the average extension of the confined WLC agreed with the simulation results very well, and those based on the classical deflection length showed an apparent discrepancy with the simulation results when the tube diameter became large or the stretching force was small.

## 3. Conclusions

Based on WLC theory and existing results on statistical properties of strongly confined polymers, we theoretically and numerically studied confinement free energy and force-confinement-extension relations of rectangular tube-confined semiflexible polymer chains under stretching in a deflection regime. We derived a modified deflection length without any adjustable parameters, which was valid for quantitative formulations of both free energy and geometrical extension. By using this deflection length scale, we obtained compact formulas on the confinement free energy and force-extension relation without any fitting parameters. Numerical analysis based on the eigenvalue problem of the governing Fokker–Planck equations and the GBR Brownian dynamics simulations justified these theoretical predictions to be valid for a much more extended range of the confinement/persistence length ratio than that based on the classical deflection length.

## Figures and Tables

**Table 1:** Prefactors of the Odijk deflection length scale.

$A_{□}$	$\alpha_{□}$
1.1036 [15]	--
1.108 ± 0.013 [17]	--
1.1038 ± 0.0006 [18]	0.09137 ± 0.00007 [18]
1.1032 ± 0.0001 [19]	0.09143 ± 0.0001 [19]

**Table 2:** Simulation parameters for the confinement of square tubes without stretching force.

Mark	Bead Radius a	Bond Length b	Time Step Δt	Contour Length L	Total Simulation Time	Total Trajectory Number
Square	0.98 nm	2 nm	10 ps	160 nm	60 μs	72
Rhombus	1.85 nm	4 nm	20 ps	320 nm	120 μs	72
Circle	0.98 nm	2 nm	10 ps	120 nm	60 μs	72
Cross	1.85 nm	4 nm	20ps	240 nm	120 μs	72

**Table 3:** Simulation parameters for the confinement of square tubes.

Confinement Size $H/L_{p}$	Bead Radius a	Bond Length b	Time Step Δt	Contour Length L	Total Simulation Time	Total Trajectory Number
0.2	1.85 nm	4 nm	10 ps	120 nm	60 μs	120
0.3	1.85 nm	4 nm	10 ps	120 nm	60 μs	120
0.4	1.85 nm	4 nm	20 ps	600 nm	120 μs	120
0.6	1.85 nm	4 nm	20 ps	600 nm	120 μs	120

**Table 4:** Simulation parameters for the confinement of rectangular tubes.

Confinement Size $H_{w}/L_{p},\;H_{h}/L_{p}$	Bead Radius a	Bond Length b	Time Step Δt	Contour Length L	Total Simulation Time	Total Trajectory Number
0.2, 0.3	2 nm	5 nm	20 ps	300 nm	120 μs	120
0.3, 0.4	1.85 nm	4 nm	20 ps	200 nm	120 μs	120
0.3, 0.6	1.85 nm	4 nm	20 ps	200 nm	120 μs	120
0.4, 0.6	2 nm	5 nm	20 ps	300 nm	120 μs	120

**Table 5:** Simulation parameters for the confinement of slits (circle).

Confinement Size $H_{h}/L_{p},\;H_{w}/L_{p}$	Bead Radius a	Bond Length b	Time Step Δt	Contour Length L	Total Simulation Time	Total Trajectory Number
0.2, 1000	2 nm	5 nm	15 ps	200 nm	90 μs	120
0.3, 1000	2 nm	5 nm	20 ps	150 nm	120 μs	120
0.4, 1000	1.85 nm	4 nm	25 ps	400 nm	150 μs	120
0.6, 1000	1.85 nm	4 nm	20 ps	400 nm	120 μs	120

**Table 6:** Simulation parameters for the confinement of slits (cross).

Confinement Size $H_{h}/L_{p},\;H_{w}/L_{p}$	Bead Radius a	Bond Length b	Time Step Δt	Contour Length L	Total Simulation Time	Total Trajectory Number
0.2, 1000	1.85 nm	4 nm	20 ps	240 nm	120 μs	120
0.3, 1000	1.85 nm	4 nm	20 ps	240 nm	120 μs	120
0.4, 1000	2 nm	5 nm	25 ps	500 nm	150 μs	120
0.6, 1000	2 nm	5 nm	25 ps	500 nm	120 μs	120

## Appendix A. The GBR Model for Brownian Dynamics Simulations

In the GBR model, a semiflexible chain is described as *N* identical virtual beads of radius *a*, which is connected by *N*−1 inextensible rods of length *b.* Each bead has a different position in a Cartesian coordinate system, $r_{k}(t)=\{x_{k}(t),y_{k}(t),z_{k}(t){\}}^{\prime }$, *k* = 1, 2, …, *N*, which is introduced for accounting hydrodynamic interactions between different chain segments. These segments are treated as hard rods, whose geometrical constraints are realized by the so-called linear constraint solver (LINCS) [33,38]. Evolution of chain configurations is characterized by a collective Brownian motion of *N* identical beads in solution. By denoting the time step, Δt, and position vectors of all the beads at the current *n* Δt, $r_{\left(n\right)}$ = {$r_{1,(n)}$, $r_{2,(n)}$, …, $r_{N,(n)}$}’, then the position vectors $r_{\left(n+1\right)}$ at (*n* + 1) Δt can be determined from the governing Langevin equation in terms of the first-order difference method as

(A1) $$r_{\left(n+1\right)}=r_{\left(n\right)}+\frac{\Delta t}{k_{B}T}D_{\left(n\right)}(F_{\left(n\right)}+B_{\left(n\right)}^{T}\lambda_{\left(n\right)})+\xi_{\left(n\right)},$$

where $D_{\left(n\right)}$ is the translational diffusion matrix consisting of 3 × 3 sub-blocks $D_{\left(n\right)}^{jk}$, *j*, *k*=1, 2, …, *N*, for which the Rotne–Prager tensor [39] is used to represent the hydrodynamic interactions between beads *j* and *k*. In a case where only equilibrium properties are interested, the sub-blocks $D_{\left(n\right)}^{jk}$ can be simply set as

(A2) $$D_{\left(n\right)}^{jk}=\frac{k_{B}T}{6\pi \eta a}\left(\begin{array}{ccc} 1 & 0 & 0\\ 0 & 1 & 0\\ 0 & 0 & 1 \end{array}\right).$$

Such a simplification will not affect various average results in the equilibrium state as long as a sufficiently large number of different configurations can be generated. In Equation (A11), $F_{\left(n\right)}$ is the collective vector, including internal bending forces and external forces; and $B_{\left(n\right)}$ is the associated gradient matrix related to the *N* − 1 time-independent constraint equations for the inextensible rods

(A3) $$g_{i,(n)}(r)=\left\|r_{i,(n)}-r_{i+1,(n)}\right\|-d_{i}=0$$

and

(A4) $$B_{\left(n\right)}=\left\{\frac{\partial g_{i,(n)}(r)}{\partial r},i=1,2,\cdots ,N-1\right\},$$

where $d_{i}$ is the length of the *i*th rod, and the dimensions of gradient matrix $B_{\left(n\right)}$ are (N−1)×3N. Vector $\lambda_{\left(n\right)}$ consists of *N* − 1 Lagrange multipliers, so that $B_{\left(n\right)}^{T}\lambda_{\left(n\right)}$ can be regarded as collective constraint forces keeping the rod length constant. The vector $\xi_{\left(n\right)}$ represents random forces generated at each time step from a Gaussian distribution with zero mean and variance

(A5) $$\langle \xi_{\left(n\right)}\xi_{\left(n^{\prime }\right)}\rangle =2D_{\left(n\right)}\Delta t\delta_{nn^{\prime }},$$

where $\delta_{nn^{\prime }}$ is the Kronecker delta symbol.

As $B_{\left(n\right)}$ is time-independent, the time derivative of Equation (A3) yields

(A6) $$\frac{dg_{\left(n\right)}}{dt}=B_{\left(n\right)}\frac{dr}{dt}=B_{\left(n\right)}\frac{r_{\left(n+1\right)}-r_{\left(n\right)}}{\Delta t}\approx 0.$$

Based on Equations (A1) and (A6), we can determine the Lagrange multipliers as

(A7) $$\lambda_{\left(n\right)}=-{\left(B_{\left(n\right)}D_{\left(n\right)}B_{\left(n\right)}^{T}\right)}^{-1}B_{\left(n\right)}\left(D_{\left(n\right)}F_{\left(n\right)}+\frac{k_{B}T}{\Delta t}\xi_{\left(n\right)}\right).$$

Inserting Equation (A7) into Equation (A1) and adding an additional term $-T_{\left(n\right)}(B_{\left(n\right)}r_{\left(n\right)}-d)$ to suppress the accumulation of numerical errors, then the new position vector can be further expressed as

(A8) $$r_{\left(n+1\right)}=(I-T_{\left(n\right)}B_{\left(n\right)})(r_{\left(n\right)}+\frac{\Delta t}{k_{B}T}D_{\left(n\right)}F_{\left(n\right)}+\xi_{\left(n\right)})+T_{\left(n\right)}d,$$

where $T_{\left(n\right)}=D_{\left(n\right)}B_{\left(n\right)}^{T}{\left(B_{\left(n\right)}D_{\left(n\right)}B_{\left(n\right)}^{T}\right)}^{-1}$, and $I-T_{\left(n\right)}B_{\left(n\right)}$ is a projection matrix that sets the constraints.

For the confinement of a rectangular tube with width and height $H_{w}$ and $H_{h}$, we used a semi-analytical technique proposed by Peters and Barenbrug [40] to treat the collective stochastic motion of *N* beads near the reflecting tube walls. Considering the *j*th bead with current position $r_{j,\left(n\right)}$, we defined $S_{j,(n)}$ as the current distance of the *j*th bead from a wall. The bead can be viewed as close enough to the tube wall as long as

(A9) $$S_{j,(n)}\leq \sqrt{5\Delta tk_{B}T/6\pi \eta a},j=1,2,\cdots ,N.$$

The influence of the tube wall on the movement of the *j*th bead in the next time step can be characterized by the random displacement $\delta r_{j,(n)}$, as derived by Peters and Barenbrug [40]:

(A10) $$\delta r_{j,(n)}=\left[f_{1}\left(\frac{S_{j,(n)}}{\sqrt{\Delta tk_{B}T/6\pi \eta a}}\right)+f_{2}\left(\frac{S_{j,(n)}}{\sqrt{\Delta tk_{B}T/6\pi \eta a}}\right)\sqrt{k_{B}T/6\pi \eta a}\Delta \zeta \right]n,$$

(A11) $$f_{1}\left(x\right)=\frac{2}{\sqrt{\pi }}\exp\left(-\frac{x^{2}}{4}\right)-x\left[1-\mathrm{erf}\left(\frac{x}{2}\right)\right],$$

(A12) $$f_{2}\left(x\right)=\sqrt{2+x^{2}-{\left[x\mathrm{erf}\left(\frac{x}{2}\right)+\frac{2}{\sqrt{\pi }}\exp\left(-\frac{x^{2}}{4}\right)\right]}^{2}},$$

where **n** is the unit normal vector of the tube wall, and Δζ is a random variable with first moment equal to 0. When $S_{j,(n)}>\sqrt{5\Delta tk_{B}T/6\pi \eta a}$, we set $\delta r_{j,(n)}=0$, implying that the bead is not sufficiently close to the wall. The stochastic displacements for all beads induced by the wall at the *n*th time step can be put in a 3*N* vector $\chi_{\left(n\right)}^{\mathrm{wall}}$. Substituting $\chi_{\left(n\right)}^{\mathrm{wall}}$ into Equation (A8) leads to

(A13) $$r_{\left(n+1\right)}=(I-T_{\left(n\right)}B_{\left(n\right)})(r_{\left(n\right)}+\chi_{\left(n\right)}^{\mathrm{wall}}+\frac{\Delta t}{k_{B}T}D_{\left(n\right)}F_{\left(n\right)}+\xi_{\left(n\right)})+T_{\left(n\right)}d.$$

If the chain is stretched by external forces, then the force vector in the above equation can be expressed as

(A14) $$F_{\left(n\right)}=F_{\left(n\right)}^{b}+F_{\left(n\right)}^{t}.$$

where $F_{\left(n\right)}^{b}$ represents the internal forces on all beads due to bending deformation [33], and $F_{\left(n\right)}^{t}$ represents the tensile forces.
